# A not-to-miss Cause of Severe Cervical Spine Pain in a Patient with Rheumatoid Arthritis: A Case-Based Review

**DOI:** 10.31138/mjr.32.3.256

**Published:** 2021-08-25

**Authors:** Alexandros A. Drosos, Eleftherios Pelechas, Athanasios N. Georgiadis, Paraskevi V. Voulgari

**Affiliations:** Rheumatology Clinic, Department of Internal Medicine, Medical School, University of Ioannina, Ioannina, Greece

**Keywords:** rheumatoid arthritis, cervical spine, conventional radiography, neck pain, joint instability, atlanto-axial subluxation, subaxial subluxation, imaging

## Abstract

**Background:**

Rheumatoid arthritis (RA) may affect any diarthrodial joint with a predilection on the peripheral skeleton in a symmetrical manner. When the axial skeleton is affected, it is the cervical spine (CS) that gets involved with potentially detrimental effects, if not treated promptly.

**Case:**

A 60-year-old female suffering from RA presented with severe neck pain and stiffness, difficulty of standing and walking with brisk tendon reflexes, Babinski sign positive, and clonus. Despite the high inflammatory markers and high titres of autoantibodies (rheumatoid factor and anticitrullinated protein antibodies), she never received proper treatment. She was using only paracetamol and non-steroidal anti-inflammatory drugs. Conventional radiography (CR) of CS showed extensive degenerative changes affecting the C3–C5 vertebral level. Magnetic Resonance Imaging of the neck showed sub-axial subluxation (SAS) and spinal cord compression at C3 level, and to a lesser extent, in other levels. A multi-level cervical laminectomy and spinal cord decompression were deployed with good results. To this end, literature review was performed until September 2020 and showed that the frequency of radiological findings varies substantially, ranging between 0,7–95% in different studies. The most common radiological feature is the atlanto-axial subluxation (AAS) followed by SAS. Because CS involvement can often be clinically asymptomatic, its assessment should not be forgotten by physicians and should be assessed using CR, which is an easy-to-perform technique and gives important information as a screening tool. On the other hand, RA patients need to be treated in a prompt and efficient manner in order to avoid any potentially fatal complications.

## INTRODUCTION

Rheumatoid arthritis (RA) is a chronic inflammatory disease affecting mainly the synovial membrane of the peripheral joints, as well as the cervical spine (CS) of the axial skeleton. It affects females more frequently than males in a ratio of 3:1 at all ages.^1,2^ The most common site of inflammation of CS is the atlanto-axial region, the articulation between C_1_ and C_2_ vertebrae. The most common radiological manifestations of CS in RA are the atlanto-axial subluxation (AAS), followed by the sub-axial subluxation (SAS), the articulations below the C_2_ vertebrae.^3,4^ Although CS involvement is a common radiological finding in RA patients, the clinical manifestations are scarce, but sometimes potentially severe and life-threatening with serious neurological complications,^5–7^ as in the case we present below.

## CASE PRESENTATION

A 60-year-old female presented to us with severe neck pain and stiffness, as well as standing and walking difficulty that had been persisting the last four days. Ten years earlier, she had been diagnosed with RA, on the basis of symmetrical polyarthritis, affecting the small joints of the hands and wrists bilaterally, high erythrocyte sedimentation rate (ESR) 68 mm/h, C-reactive protein (CRP) 49 mg/dl, positive rheumatoid factor (RF) 680 U, and positive anticitrullinated protein antibodies (ACPA) 410 U.^8^

**Figure 1.**
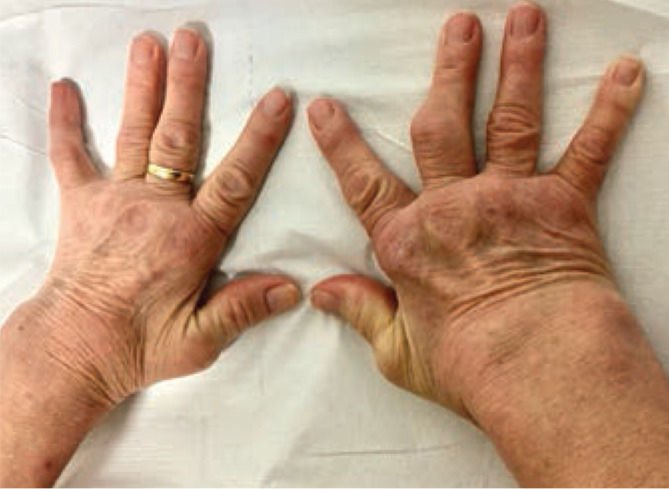
Longstanding RA findings in a 60-year-old patient. The findings are more pronounced on the right hand (dominant).

She reported that she did not receive any conventional synthetic disease-modifying antirheumatic drugs (csDMARDs), nor biological (b) DMARDs except for paracetamol, and, occasionally, non-steroidal anti-inflammatory drugs (NSAIDs). Past medical and family history were unremarkable. Clinical examination showed swelling and tenderness of the metacarpophalangeal (MCP) and proximal interphalangeal (PIP) joints bilaterally, as well as muscle atrophy (**Figure 1**). Clinical evaluation of the CS showed severe neck pain and stiffness at any head movement. Neurological examination revealed brisk tendon reflexes, symmetrical on the upper and lower extremities, and Babinski sign along with clones on the right foot. Laboratory tests revealed anaemia of chronic disease (ACD, Hb 9 g/dl, serum ferritin 158 mg/dl and serum ferrum 7 mg/dl), elevated acute phase reactants and high titres of RF and ACPA antibodies.

**Figure 2.**
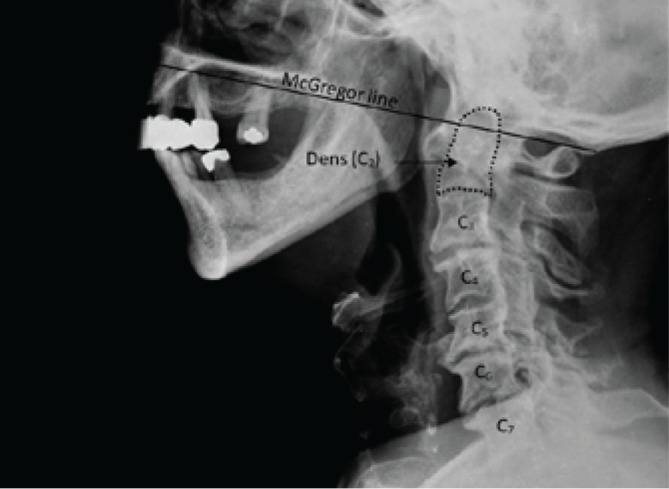
Conventional radiography of the neck - Lateral view (and after the interpretation of the image - Extensive degenerative changes involving the C3, C4 and C5 vertebral bodies, as well as fusion of the apophyseal joints C2–C3 and C4–C5.

**Figure 3.**
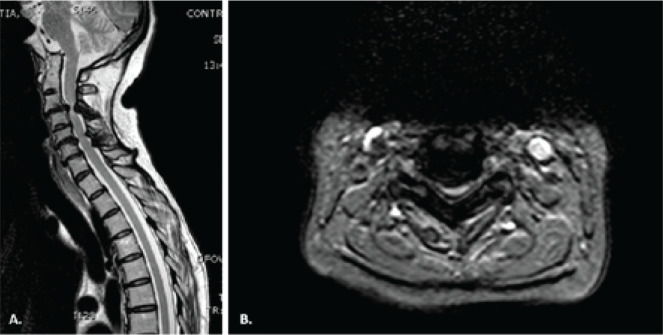
MRI of the neck. Subaxial subluxation (A - sagittal plane) and spinal compression at the C3 level (B - axial plane).

**Figure 4.**
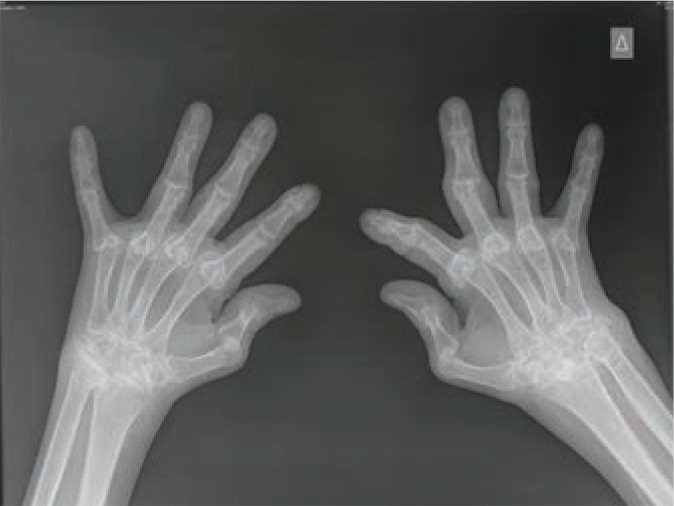
Hand x-rays. Note the severe erosive changes and subluxations of the MCP joints bilaterally, fusion of the carpal bones, and severe osteopenia.

Radiological evaluation of the CS disclosed extensive degenerative changes involving the C_3_, C_4_ and C_5_ vertebral bodies, as well as fusion of the apophyseal joints C_2_,C_3_ and C_4_,C_5_ (**Figure 2**). Magnetic resonance imaging (MRI) showed SAS and spinal cord compression at C_3_ level and to a less extent in other levels (**Figure 3A,B**). Hand x-rays showed severe erosive changes and subluxations of the MCP joints bilaterally, fusion of the carpal bones and severe osteopenia (**Figure 4**). The patient was admitted and directed to the neurosurgery department and underwent multi-level cervical laminectomy and spinal cord decompression with excellent results. Before discussing our case, we review the CS anatomy, its imaging evaluation, the role of conventional radiography (CR), the radiographic changes of CS, and the studies investigating the CS involvement in the setting of RA patients.

## ANATOMY AND RADIOGRAPHIC FINDINGS OF THE CS IN RA PATIENTS

For a better understanding of the findings in the CS of a patient with RA, the basic anatomic features are presented. Furthermore, for the evaluation of the CS in RA the classical diagnostic technique used mostly is conventional radiography (CR).^3,4^ On the other hand, CR does not provide good information regarding synovial inflammation or other soft-tissue structural changes. Thus, other imaging modalities are used, such as MRI and computed tomography (CT). MRI demonstrates active synovitis of the odontoid process, or pannus formation and erosions. Finally, a CT scan may visualize better the erosive changes of the disease.^3,4^ However, CR is the most valuable tool for screening the CS in RA patients. It is an easy-to-perform technique and gives important information about CS involvement.^5^ We reviewed the literature until December 2019 for studies regarding CS radiological manifestations in RA patients. In this review, we will discuss the value of CR as a screening tool for the evaluation of the CS and the radiological findings occurring in this setting.

### Cervical spine anatomy

**Figure 5.**
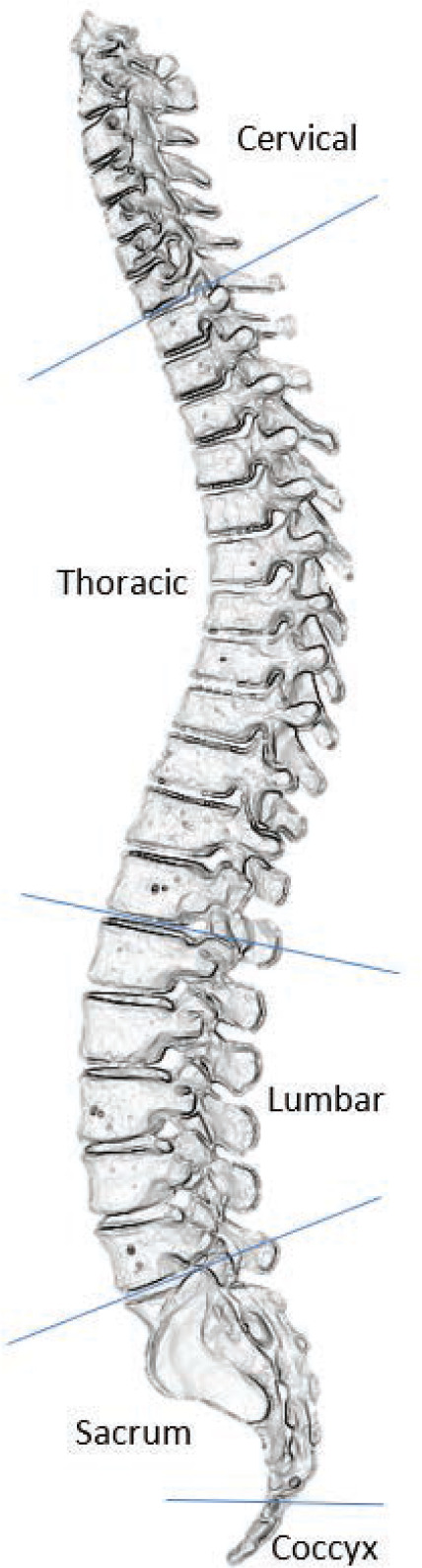
Vertebral spine and its anatomic divisions.

**Figure 6.**
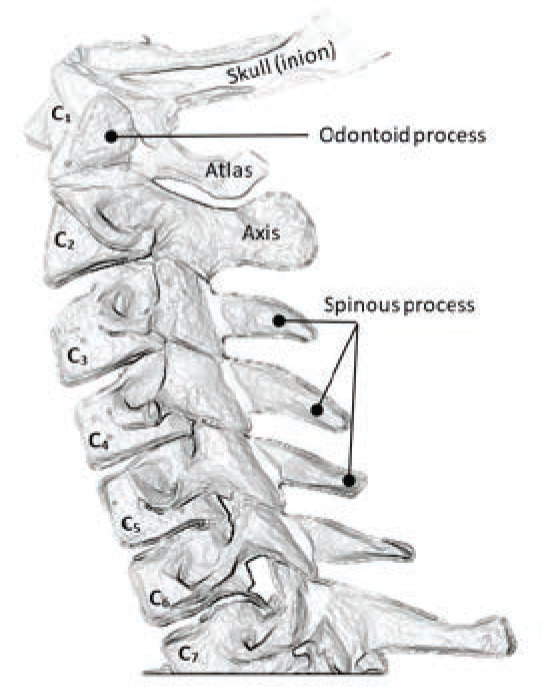
Anatomy of the cervical spine (lateral view).

The spinal (or vertebral) column is part of the axial skeleton and is divided in five anatomic regions: cervical spine, thoracic spine, lumbar spine, sacrum, and coccyx (**Figure 5**). CS is composed by seven cervical vertebrae from C1 to C7 (cranial to caudal), from the base of the skull (C1) down to the top of the shoulders (C7). Vertebrae present anatomic variations among them. The topmost vertebrae, and especially the C1 (atlas) and the C2 (axis), are more mobile than the lower. Atlas and axis are smaller and have a unique role allowing movements such as flexion, extension, lateral flexion, and rotation. The lower part of the cervical spine is thicker in order to handle greater loads from the neck and head (**Figure 6**). Atlas is the only vertebra without a vertebral body. It is an atypical, ring-shaped vertebra articulating to the occipital bone in order to support the base of the skull forming the atlanto-occipital joint. The second cervical vertebra (C2) has a large bony protrusion, the odontoid process or dens, that extends upward from its vertebral body and fits into the atlas forming the atlanto-axial joint.

Unlike other vertebral joints, this joint does not have an intervertebral disc. The dens is held in place by a thick strong ligament, the transverse ligament, and by the alar and apical ligaments. The rest of the CS vertebrae bellow C2 are known as typical vertebrae because they share the same basic characteristics with the other vertebrae of the spine. They separate between them by an intervertebral disc.^9,10^

### Radiological evaluation of CS in RA patients

**Figure 7.**
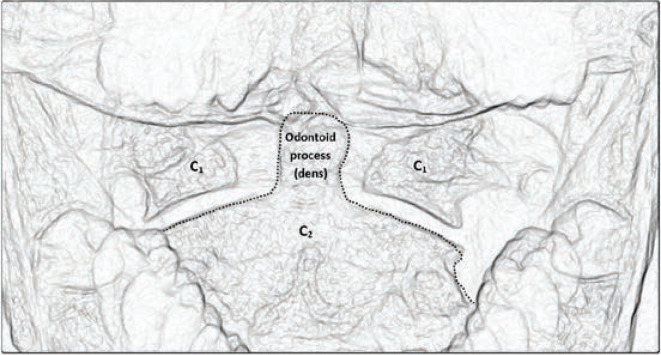
Open-mouth x-ray. Schematic representation.

CR, iis a useful screening tool in RA patients giving important information for any cervical instability. Antero-posterior (AP), upright, lateral, flexion, and extension views can be used in the radiological evaluation of the CS. In addition, an open-mouth x-ray can be used for the evaluation of the odontoid process (**Figure 7**). When abnormalities are suspected or confirmed with CR, then other imaging modalities such as MRI or CT scan must be performed. MRI is the most sensitive imaging modality for the detection of active synovitis of the odontoid process, pannus formation, ligament laxity, and erosions. On the other hand, CT scan with multiple projection reconstruction (MPR) is superior in demonstrating any erosive changes.^3,4^

The atlanto-axial region is the most common site of inflammation of the CS, and more specifically, between the articulation of the C1 and C2 vertebrae. Weakening of the structures or rupture of ligaments as well as subchondral bone erosions may lead to AAS instability which is the mostly observed radiological finding in the CS region of the spine.^11–13^ SAS of the CS is defined as the segment bellow the C2 vertebra, that is from C3 to C7. Other CS abnormalities comprise: upper disc space narrowing, vertebral plate erosions and sclerosis as well as apophyseal joints erosions and sclerosis. CS abnormalities as described above are frequent radiographic findings in RA patients, but the clinical features are scarce and minimal but potentially life-threatening. One of the most common and with underlying risk radiographic finding in CS is AAS.^5,6,14^

### Radiological findings of AAS

The atlanto-axial joint can be subluxed in multiple directions, leading to cervical cord compression and cause myelopathy.^4,11,14^ The atlas can move anteriorly, posteriorly, laterally, vertically, or rotationally related to the odontoid process of the axis. More specifically, there is an articulation between the transverse ligament of the atlas and the posterior aspect of the odontoid process. This thick and strong articulation acts as a sling in maintaining the odontoid process against the posterior surface of the atlas constant, and preventing forward movement of C1 on C2 vertebrae. Persistent inflammation of this articulation may produce dens erosions, damage of the transverse, alar and apical ligaments, and laxity leading to joint instability.^3,4,7^ The distance between the anterior aspect of the odontoid process and the posterior surface of the anterior arch of the atlas usually measures ≤3mm. If this distance increases and exceeds more than 8mm the chance of CS cord compression is high. However, the posterior atlanto-dental distance has been found to be a better predictor for cord compression. Indeed, the distance from the posterior border of the dens to the anterior aspect of the posterior arch of the C1 vertebra, represents the maximum amount of space for the CS cord. In detail: in CS the cord occupies 10mm of the canal diameter, requires 1mm for the dura and 1mm for the CS fluid anterior to the cord, and 1mm posteriorly. Thus, the total space is 14mm. If the available space becomes <14mm, then CS cord becomes compressed. Thus, in AAS if the anterior atlanto-dental distance increases more than 3mm and the posterior atlanto-dental distance decreases less than 14mm, then the CS cord is prone to compression.^7,15^

### Radiological findings of the lateral AAS

Lateral AAS is rare, resulting in a rotational deformity. For a better evaluation, the open-mouth view is preferred. If any asymmetry or lateral displacement of the atlas on the axis by >2mm or an asymmetrical collapse of the lateral mass takes place on an open-mouth radiographic view, then lateral AAS must be suspected.^3,4^

### Radiological findings of the vertical AAS

Vertical AAS, also known as basilar impression on cranial setting, is a superior migration of the odontoid process, resulting in brainstem compression by the dens and/or the pannus itself. It may cause stroke, obstructive hydrocephalus, heart arrest and sudden death.^16^ Vertical AAS is present if the tip of the dental peg lays >4.5mm above the McGregor line.^17^ This is a hypothetical line drawn between the hard palate and the most caudal point of the occipital curve (**Figure 2**).

### SAS in CS in RA patients

SAS is the second most common form of CS instability in RA patients affecting the C3 to C7 vertebrae. In this type of instability, inflammation of the apophyseal joints, the intervertebral disc and the interspinous ligaments, ensues. SAS may be an isolated finding but it can involve multiple levels leading to the characteristic “staircase” deformity. SAS and AAS may appear with late neurological complications. It may also occur simultaneously. In this case severe neurological consequences may prove fatal.^13,18^ It is of interest that of a significant number of RA patients with radiographically detectable CS abnormalities, only a small number will develop CS myelopathy or other neurological complications.

## DISCUSSION

The diagnosis of CS involvement among RA patients is extremely important because it is associated with high morbidity and mortality.^19,20^ Because CS involvement can often be clinically asymptomatic, its assessment should not be forgotten by physicians. However, the American College of Rheumatology (ACR) and European League Against Rheumatism (EULAR) recommendations, as to when to evaluate the CS in RA, are missing.^21^ Most of the studies are describing patients with CS involvement as a late manifestation during the disease course and in some cases as the presenting symptom.^11,19,22–79^ The majority of them are cross-sectional or retrospective and only few in a prospective design. RA disease duration was high ranged between 2.5–30.1 years (approx. 12.3 years on average). The incidence of CS involvement ranged between 0.7 in Turkey^70^ and 95% in China,^64^ and the CS abnormalities were assessed using CR. The most common radiological features were AAS, followed by SAS. Symptoms ranged from asymptomatic to localised head and neck pain with stiffness, and a few presented neurological manifestations. The majority of RA patients were seropositive, while a few were seronegative. The diagnosis of CS involvement in RA requires a detailed questionnaire for symptoms, minute musculoskeletal and neurological examination, and radiological assessment with CR as a screening test. Usually, there is a discrepancy between the clinical symptoms of CS involvement and the radiological abnormalities occurring in this setting. Only one study of RA patients with CS disease showed correlation between clinical symptoms, neurological manifestations and radiological damage.^80^ In the absence of clinical symptoms, if AAS or SAS or atlanto-axial impaction are present in the radiological assessment, then attention is required for surgical consultation.^15,81^

The present case describes an RA patient with a long-standing, seropositive untreated disease who later developed SAS and spinal cord compression. SAS may develop as the first radiological manifestation of CS in RA or consequently, from prior fusion of AAS at C_1_–C_2_ levels. Our patient underwent multi-level cervical laminectomy and spinal cord decompression with excellent results.^81,82^ Two weeks after her surgical operation, she was treated with methotrexate (15 mg/week) and prednisone (10 mg/day). Two months later, she presented substantial clinical and laboratory improvement, without any signs of neurological manifestation, and prednisone was tapered. This case teaches us that RA is a chronic inflammatory disease and, if left untreated, may lead to a catastrophic course, especially to patients with unfavourable prognostic factors. Indeed, our patient suffered from a long-standing, seropositive disease, with ACD and elevated acute phase reactance, without receiving any treatment. All the above are considered poor prognostic factors and are associated with radiological damage progression and disease complications.^83–86^ Thus, early and intensive intervention^87^ with close follow-up and monitoring are imperative to control the disease’s activity and avoid RA complications.
